# Evaluation of circulating microRNA profiles in blood as potential candidate biomarkers in a subacute ruminal acidosis cow model - a pilot study

**DOI:** 10.1186/s12864-023-09433-y

**Published:** 2023-06-16

**Authors:** O. E. Ojo, L. Hajek, S. Johanns, C. Pacífico, A. Sener-Aydemir, S. Ricci, R. Rivera-Chacon, E. Castillo-Lopez, N. Reisinger, Q. Zebeli, S. Kreuzer-Redmer

**Affiliations:** 1 Christian Doppler Laboratory for Innovative Gut Health Concepts of Livestock, Institute of Animal Nutrition and Functional Plant Compounds, University of Veterinary Medicine Vienna, Vienna, Austria; 2 Nutrigenomics Unit, Institute of Animal Nutrition and Functional Plant Compounds, University of Veterinary Medicine Vienna, Vienna, Austria; 3 Biome Diagnostics GmbH, Vienna, Austria; 4 DSM, BIOMIN Research Center, Tulln an Der Donau, Austria

**Keywords:** MicroRNAs, Dairy cows, Bovine, miRNA biomarkers, Subacute ruminal acidosis (SARA), High-grain feeding, Plasma, Leucocytes

## Abstract

**Background:**

Subacute ruminal acidosis (SARA) is a metabolic disorder often observed in high-yielding dairy cows, that are fed diets high in concentrates. We hypothesized that circulating miRNAs in blood of cows could serve as potential candidate biomarkers to detect animals with metabolic dysbalances such as SARA. MicroRNAs (miRNAs) are a class of small non-coding RNAs, serving as regulators of a plethora of molecular processes. To test our hypothesis, we performed a pilot study with non-lactating Holstein–Friesian cows fed a forage diet (FD; 0% concentrate, *n* = 4) or a high-grain diet (HG; 65% concentrate, *n* = 4) to induce SARA. Comprehensive profiling of miRNA expression in plasma and leucocytes were performed by next generation sequencing (NGS). The success of our model to induce SARA was evaluated based on ruminal pH and was evidenced by increased time spent with a pH threshold of 5.8 for an average period of 320 min/d.

**Results:**

A total of 520 and 730 miRNAs were found in plasma and leucocytes, respectively. From these, 498 miRNAs were shared by both plasma and leucocytes, with 22 miRNAs expressed exclusively in plasma and 232 miRNAs expressed exclusively in leucocytes. Differential expression analysis revealed 10 miRNAs that were up-regulated and 2 that were down-regulated in plasma of cows when fed the HG diet. A total of 63 circulating miRNAs were detected exclusively in the plasma of cows with SARA, indicating that these animals exhibited a higher number and diversity of circulating miRNAs. Considering the total read counts of miRNAs expressed when fed the HG diet, differentially expressed miRNAs ( log_2_ fold change) and known function, we have identified bta-miR-11982, bta-miR-1388-5p, bta-miR-12034, bta-miR-2285u, and bta-miR-30b-3p as potential candidates for SARA-biomarker in cows by NGS. These were further subjected to validation using small RNA RT-qPCR, confirming the promising role of bta-miR-30b-3p and bta-miR-2285.

**Conclusion:**

Our data demonstrate that dietary change impacts the release and expression of miRNAs in systemic circulation, which may modulate post-transcriptional gene expression in cows undergoing SARA. Particularly, bta-miR-30b-3p and bta-miR-2285 might serve as promising candidate biomarker predictive for SARA and should be further validated in larger cohorts.

**Supplementary Information:**

The online version contains supplementary material available at 10.1186/s12864-023-09433-y.

## Background

MicroRNAs are a class of small non-coding RNAs which regulate a wide range of biological processes such as mammary gland development [12, 16, 17, 20] and ovary development [46, 49], differentiation, apoptosis, and viral infection through post-transcriptional regulation of gene expression [4]. Since the first miRNA was identified in lin-4 (Caenorhabditis elegans) in 1993, an increasing number of miRNAs have been found in animals as sequencing and bioinformatics technologies advanced and ushered in a new era and the capability to quickly detect many classes of short RNA molecules, including miRNAs, in a variety of biological samples like plasma, blood cells, milk, urine, etc. [31]. With a view to comprehend the regulatory network of miRNAs and gene expression, it is crucial to understand the identification and characterization of miRNAs and their targets in animals in recent years [18]. The finding of circulating miRNAs in bodily fluids like milk, urine, and saliva [8] opened the door to the field of biomarker discovery, which uses a mix of omics technologies to achieve the least invasive detection of molecules feasible, for example in cancer biology. A miRNA biomarker is a miRNA that is generated or enriched in a specific tissue, and whose circulating levels may reflect pathological or physiological changes in that tissue associated to diseases or progression of diseases. MiRNAs have recently become highly useful biomarkers of human infectious, genetic, and metabolic diseases but there are only a few studies in farm animals. In the past few years, profiles of circulating miRNAs have been connected to pregnancy and the oestrous cycle [21, 22], infection [11, 15], and various physiological processes, metabolic changes, and adaptive response pathways driving skeletal muscle growth in cattle [44].

Subacute ruminal acidosis (SARA) is a frequent metabolic disorder of high-producing cows, commonly associated with a high-grain (HG) diet and is identified by a drop of ruminal pH due to rapid accumulation of volatile fatty acids, as well as lactic acid [42, 59]. Cows experiencing SARA undergo both ruminal and systemic inflammation, which are induced by a cascade of events starting with dysbiosis in the rumen and resulting in the activation of the innate immune response and several metabolic disorders [7, 60].

In this study, we hypothesized that blood miRNAs could serve as potential candidate biomarkers for rumen health in cows using a diet-induced SARA bovine model. To test our hypothesis, comprehensive identification of miRNA profiles were conducted and miRNAs in plasma and leucocytes were evaluated towards its potential to serve as candidate biomarkers in cows that were exposed to SARA risk by a HG feeding period consisting of a 65% concentrate diet. The presence of specific miRNAs in the plasma and leucocytes could contribute to understand the systemic pathophysiology of SARA in cattle. In addition, miRNAs could serve as valid biomarkers to identify cows suffering from SARA in a herd. Furthermore, biomarkers could be used to identify those cows which are able to better cope with SARA, also considering further breeding strategies. The findings of our study contribute to a better understanding of how dietary changes affect the systemic circulation of miRNAs and provide data for miRNAs and their potential use as biomarkers of SARA in cows.

## Results

### MiRNA expression pattern in plasma and blood leucocytes

**Fig. 1:**
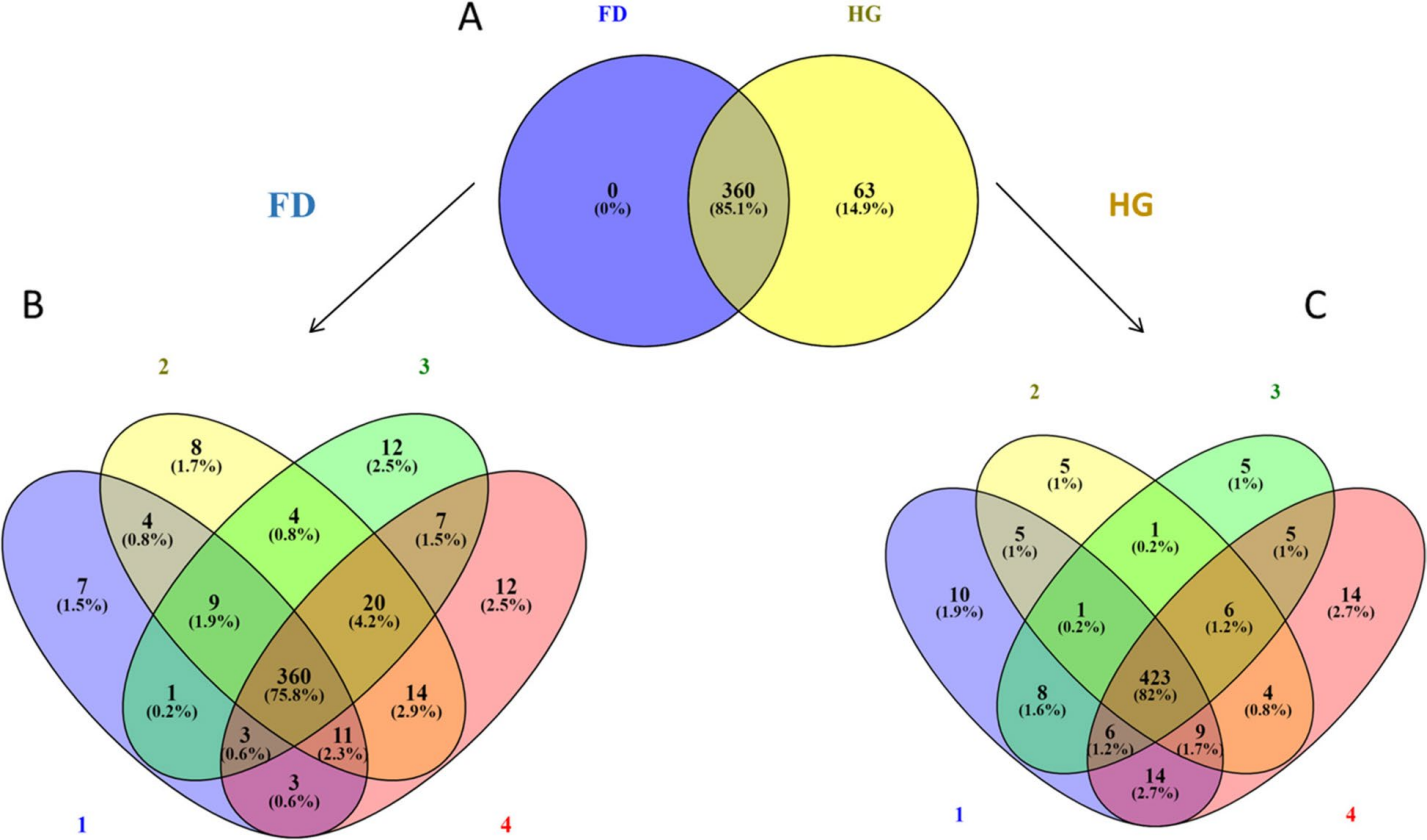
Venn diagram of microRNAs expressed in plasma. **A** Zero record of miRNAs expressed in forage-based diet leucocytes and 63 miRNAs exclusively expressed in high-grain diet in plasma. **B** 360 miRNAs shared within all cows (1, 2, 3 and 4) on forage-based diet (**C**) 423 miRNAs shared within all cows (1, 2, 3 and 4) on high-grain diet

We evaluated whether our cow model would successfully induce SARA in cows. We discovered that 100% of the cows in our cow model experienced at least episodes of SARA, which were indicated by increased time spent with a pH threshold of 5.8 for an average length of 320 min/d. In total, 520 miRNAs were found in plasma (Fig. 1a, b, c), with 360 of those shared by all forage diet (FD) and HG diets (Fig. 1a). We examined all the miRNAs found in the plasma from the four cows fed on FD and later a HG diet, which resulted in the identification of 398, 430, 416, and 430 miRNAs in each cow (Fig. 1b and c). Hence, for FD, there were 423 individual cow miRNAs on HG and 360 shared miRNAs between all cows. For additional analysis, the shared miRNAs for FD and HG were examined (Fig. 1a), and none was found to be distinct to cows on FD diet, whereas 360 miRNAs were shared between the FD AND HG diet (Fig. 1a). We further discovered that several miRNAs in the cows' plasma were exclusively expressed when they were fed an HG diet, which suggests that the HG diet causes a higher level of circulating miRNAs. As a result, we recorded 63 miRNAs from the cows exclusively when they were fed the HG diet (Fig. 1a), then further sorted, and filtered them according to total read counts, read counts across animals, and known function.

**Fig. 2:**
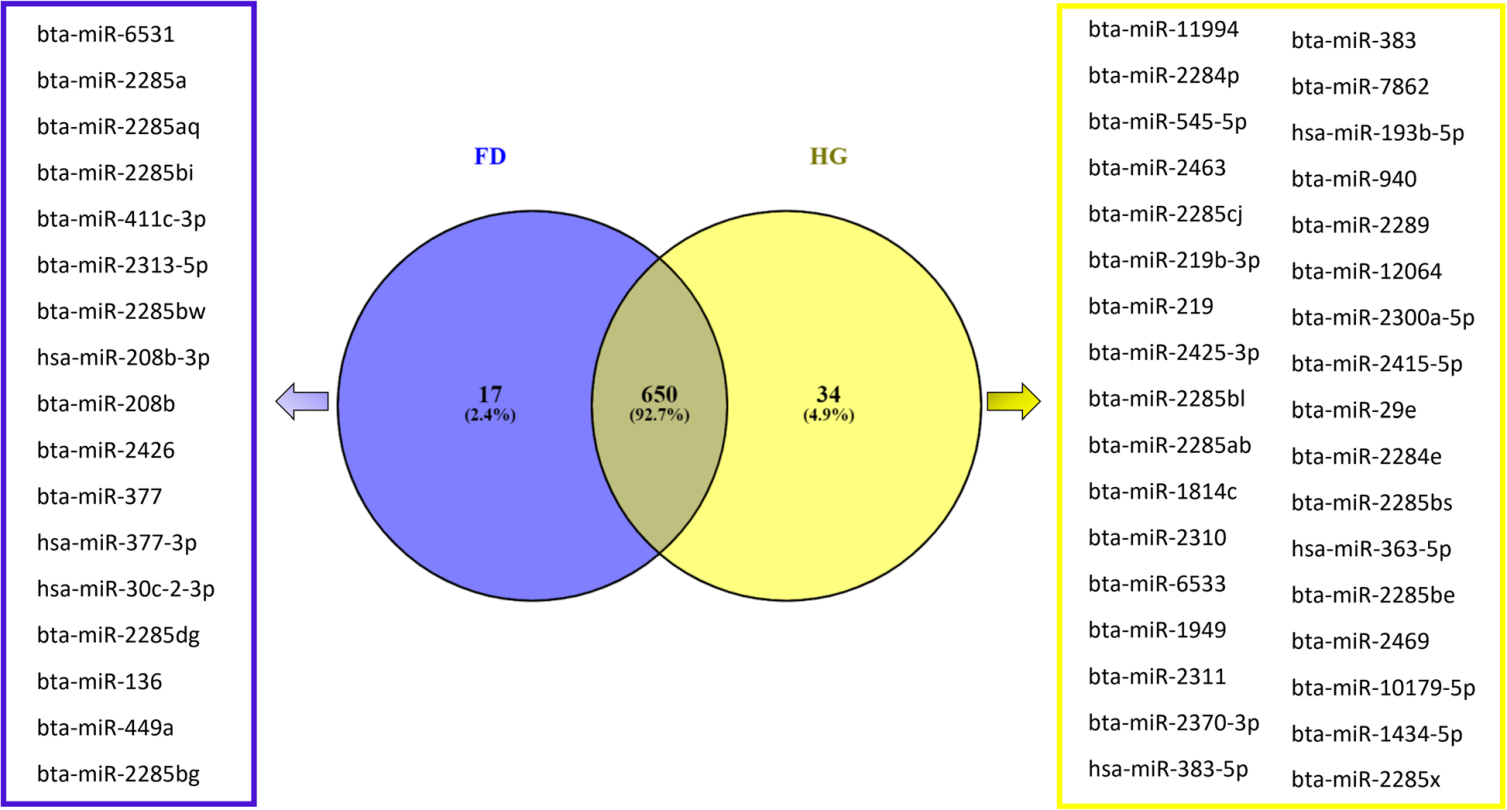
Venn diagram miRNAs expressed in leucocytes. 17 miRNAs were exclusively expressed in forage-based diet and 34 miRNAs were exclusively expressed in high-grain diet

In total, 730 miRNAs were found and recorded in leucocytes, with 650 miRNAs shared by all analysed samples (Fig. 2), which could be considered as a core microRNAome within our dataset. When comparing FD and HG diets, 17 miRNAs were found to be expressed solely on the FD, while 34 miRNAs on the HG diet.

By analyzing the unique miRNAs present in every cow and diet, we were able to determine the animal-specific miRNAs. As a result, miRNAs identified as unique and expressed in cows fed on both diets were chosen. Three cows were also found to have three animal-specific miRNAs: bta-miR-2285bb, bta-miR-664a, and bta-miR-2285am-5p (leucocytes).

**Fig. 3:**
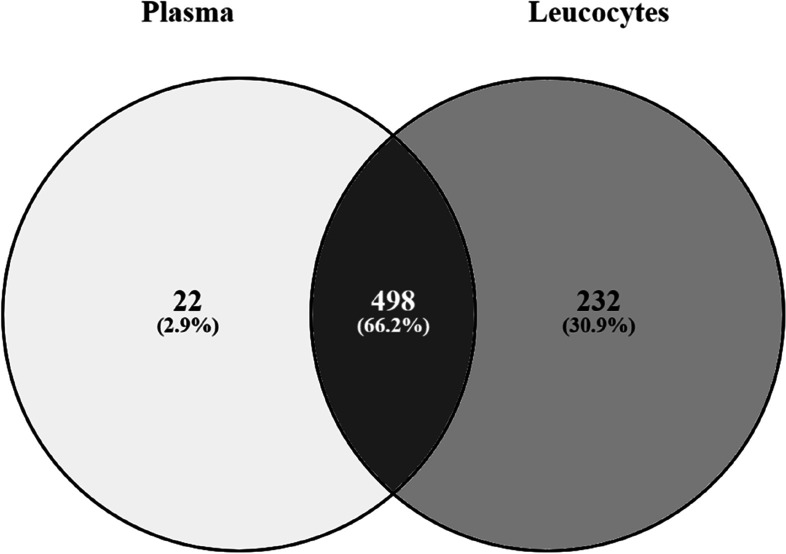
Venn diagram of microRNAs shared between plasma and leucocytes. 22 miRNAs were exclusively expressed in plasma while 232 miRNAs were exclusively expressed in leucocytes and a total of 498 miRNAs were shared between plasma and leucocytes

An inspection of the whole dataset identified 498 miRNAs shared between plasma and leucocytes (Fig. 3). In addition, 22 miRNAs were expressed solely in plasma and 232 other miRNAs in leucocytes (Fig. 3). Hence, we observed a higher number of miRNAs in leucocytes than in plasma. The ten most abundant miRNAs (Table 1) reported to be shared between plasma and leucocytes have been ranked by decreasing read counts from the 498 shared miRNAs identified between plasma and leucocytes.

**Table 1:** The 10 most expressed miRNAs in plasma and leucocytes

**Plasma**	**Leucocyte**
hsa-miR-451a	bta-miR-451
bta-miR-451	hsa-miR-451a
bta-miR-21-5p	bta-miR-21-5p
bta-mir-143	Hsa-mir-16-5p
bta-let-7a-5p	Bta-mir-16b
bta-let-7f	Bta-mir-26a
hsa-let-7f-5p	Hsa-mir-26a-5p
bta-mir-26a	Bta-mir-486
hsa-mir-26a-5p	Hsa-mir-486-5p
bta-mir-24-3p	Bta-let-7f

### Feeding a SARA inducing high grain diet affects the expression of miRNAs in plasma and blood leucocytes

**Fig. 4:**
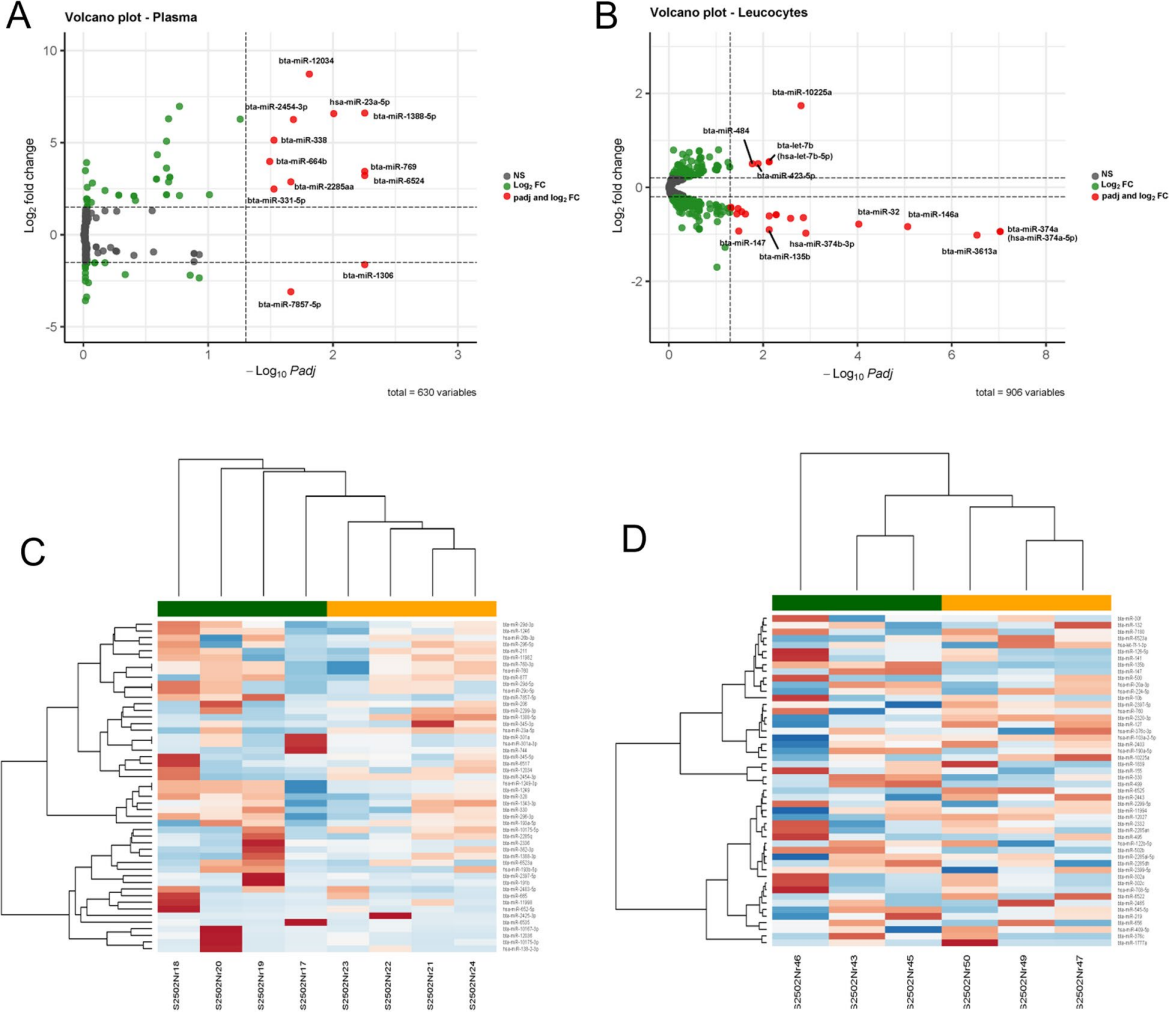
MicroRNA expression levels represented by a volcano plot (**A**) in plasma, (**B**) in leucocytes, and miRNA expression levels represented by a heat map (**C**) in plasma and (**D**) in leucocytes. In (**A**) and (**B**) each dot represents a single miRNA with red points indicating 10 upregulated and 2 downregulated miRNAs. The green points denote the log2 foldchange while the grey points indicated the non-significant. Plots were created with R software. In (**C**) and (**D**) green and orange represent forage and high-grain feeding, respectively, while red and blue indicate upregulated and downregulated miRNAs

**Fig. 5:**
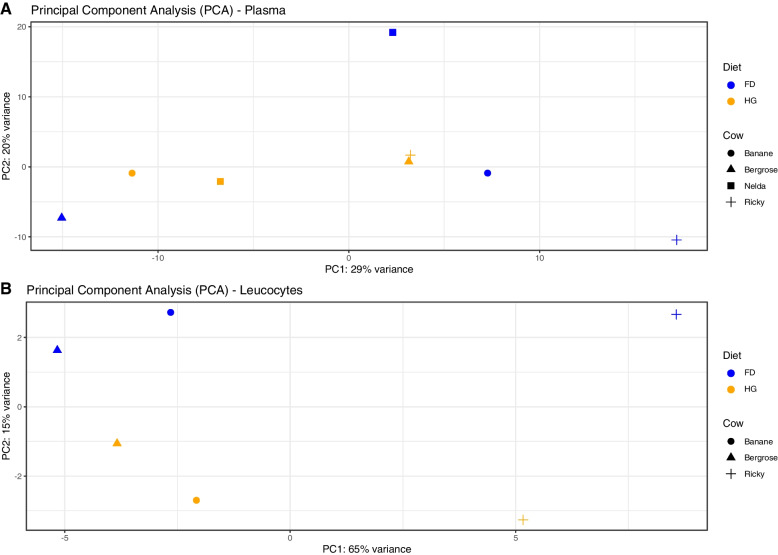
Principal component analysis of miRNA composition in (**A**) plasma, showing variation between diets and cows, and in (**B**) leucocytes, showing variation between diets and cows: blue; forage diet, orange; high-grain diet (65% grain)

Differential expression analysis revealed that 12 miRNAs were differentially expressed in plasma samples (FD vs. HG diet) with a false discovery rate (FDR) lower than 0.05 (Table 2), from which 10 miRNAs (bta-miR-12034, has-miR-23a-5p, bta-miR-2454-3p, bta-miR-1388-5p, bta-miR-338, bta-miR-664b, bta-miR-2285aa, bta-miR-6524, bta-miR-331-5p, bta-miR-769) were found to be up-regulated when the cows were fed a HG diet and 2 miRNAs were down-regulated (bta-miR-1306, bta- miR-7857-5p) (Fig. 4a). With an FDR < 0.05, 25 miRNAs were differentially expressed in leucocytes (forage vs. HG diet), of which 5 miRNAs (bta-miR-10225a, bta-miR-484, bta-let-7b, (hsa-let-7b-5p), bta-miR-423-5p) were up-regulated when the cows were fed the HG diet and 20 miRNAs were found to be down-regulated (Fig. 4b). In plasma as well as in leucocytes samples, hierarchical clustering detected a separation based on similarity of miRNA expression in the two different diets (Fig. 4c and d, respectively). The clustering of the samples was also investigated using principal component analysis (PCA) of miRNA read counts adjusted to the unique mappings in the bovine genome. Within plasma samples the first PC (PC1) was responsible for 29% of the variance between samples, whereas the second PC (PC2) was responsible for 20% (Fig. 5a). PC1 shows a cluster for the HG diet, whereas for forage only three out of the four animals clustered together. For plasma samples, PC1 seems to explain differences regarding the diet within the same four animals.

**Table 2:** 12 Differentially expressed (DE) miRNAs in plasma (FDR < 0.05)

**miRNA**	**baseMean**	**log**_**2**_**FoldChange**	**lfcSE**	**stat**	***p*** **value**	**padj**
bta-miR-1306	1502.777	-1.6245	0.400529	-4.05588	4.99E-05	0.005573
bta-miR-6524	52.5201	3.216403	0.787365	4.085023	4.41E-05	0.005573
bta-miR-769	47.04335	3.437863	0.853275	4.029021	5.60E-05	0.005573
bta-miR-1388-5p	38.93589	6.606347	1.554278	4.250429	2.13E-05	0.005573
hsa-miR-23a-5p	65.38827	6.574441	1.712976	3.838023	0.000124	0.009873
bta-miR-12034	113.5294	8.72227	2.370254	3.679889	0.000233	0.015478
bta-miR-2454-3p	143.8439	6.253793	1.754627	3.564172	0.000365	0.020753
bta-miR-2285aa	42.08954	2.87036	0.822806	3.488501	0.000486	0.021773
bta-miR-7857-5p	78.9668	-3.09793	0.888963	-3.48488	0.000492	0.021773
bta-miR-331-5p	50.22548	2.482085	0.739508	3.356399	0.00079	0.029727
bta-miR-338	33.27151	5.134805	1.53488	3.345413	0.000822	0.029727
bta-miR-664b	32.2357	3.977138	1.205475	3.299229	0.00097	0.032155

Within blood leucocytes, PC1 explains 65% of variation and clearly distinguishes the animals, and PC2 separates the diets with a 15% variance (Fig. 5b). Three of the most up-regulated differentially expressed miRNAs between the diets were chosen for target prediction (Table 3).

**Table 3:** Target prediction [54] of differentially expressed circulating miRNAs

**miRNAs**	**Total no. of transcript found**	**Important immune associated target genes**	**Predicted target sites**
bta-miR-30b-3p	4313	CD28	4
		CD160	3
		CD180	2
bta-miR-2285u	6100	IGSF6	1
		CD300LG	2
		CD164	2
bta-miR-1388-5P	2715	CD84	2
		CD86	1
		CD300LB	1

### Identification of potential candidate biomarkers

Due to the strong effect of diet on the miRNA expression profile in plasma and since plasma is a relatively easily available sample matrix, we further focused on miRNAs expressed in plasma to identify potential candidate biomarkers of SARA in our cow model. To validate the NGS data, small RNA RT-qPCR assays were established and performed for the most promising biomarker candidates. We carefully selected 6 miRNAs (bta-miR-2285u, bta-miR-30b-3p, bta-miR-12034, bta-miR-11982, bta-miR-1306, and bta-miR-1388-5p) (Table 4) for further in-depth analyses, based on the total read counts of the filtered miRNAs present in all the cows fed a HG diet (and not expressed in the FD) (*n* = 63), as well as the differentially expressed miRNAs (*n *= 12) in plasma samples. References to previous literature, as well as the functions annotation on miRBase 22 (www.mirbase.org [26]) were also considered for the selection of potential candidate biomarkers (Table 4). To further understand the biological functions of the selected miRNAs for further validation, we carried out the target predictions of the upregulated miRNAs in plasma. To monitor the expression of the potential candidate biomarkers for a longer period we included a third time point from the same cows, when cows were fed the HG diets for three weeks (HG3). To complete the picture, we measured those selected miRNAs based on its expression pattern in plasma samples, also in leucocyte samples by small RNA RT-qPCR.

**Table 4:** Selected potential candidate biomarkers

**miRNAs**	**Sequence**	**Detected in other studies**
**bta-miR-1306**	CCACCTCCCCTGCAAACGTCC (21 bp)	Strozzi et al., 2009 [50]
**bta-miR-11982**	TTCGGCGCCACCACCCTGCGGGT (23 bp)	Li et al., 2016 [34]
**bta-miR-1388-5p**	AGGACTGTCCAACCTGAGAAT (21 bp)	Li et al., 2015; [33] Ioannidis & Donadeu, 2018 [23]; Capra et al., 2017 [3]
**bta-miR-12034**	CCCCGGGGAGCCCGGCGGT (19 bp)	Li et al., 2015 [33]
**bta-miR-2285u**	GAAAAACCCGAACGAACTTT (20 bp)	Fang et al., 2018; [10] Salilew-Wondim et al., 2014 [47]; Guan et al., 2017 [14]; Lawless et al., 2013 [29]
**bta-miR-30b-3p**	TTCATTTGTAGGTGGAGGGTC (21 bp)	Zhang et al., 2016 [61]; Muroya et al., 2016 [39]; Jin et al., 2014 [24]. Sun et al., 2015 [52]; Chen et al., 2014 [6]; Capra et al., 2017 [3]; Wang et al., 2016 [56]; Fang et al., 2018 [10]

**Fig. 6:**
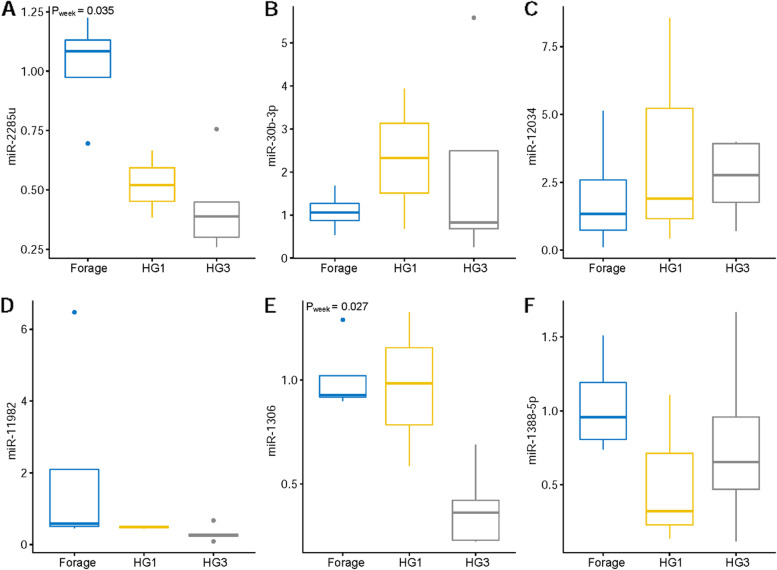
RT-qPCR validation of selected miRNAs as potential biomarker candidates in plasma. The miRNAs were selected based on NGS results (**A**) miR-2285u for high read counts, (**B**) miR-30b-3p for high expression with HG, (**C**) miR-12034 for up-regulation with HG, (**D**) miR-11982 for for high read counts, (**E**) miR-1306 for down-regulation with HG, (**F**) miR-1388-5p for up-regulation with HG. MiR-103 and miR-107 were used as internal standard for normalization. HG1 = First week of high-grain feeding, HG3 = Third week of high-grain feeding

**Fig. 7:**
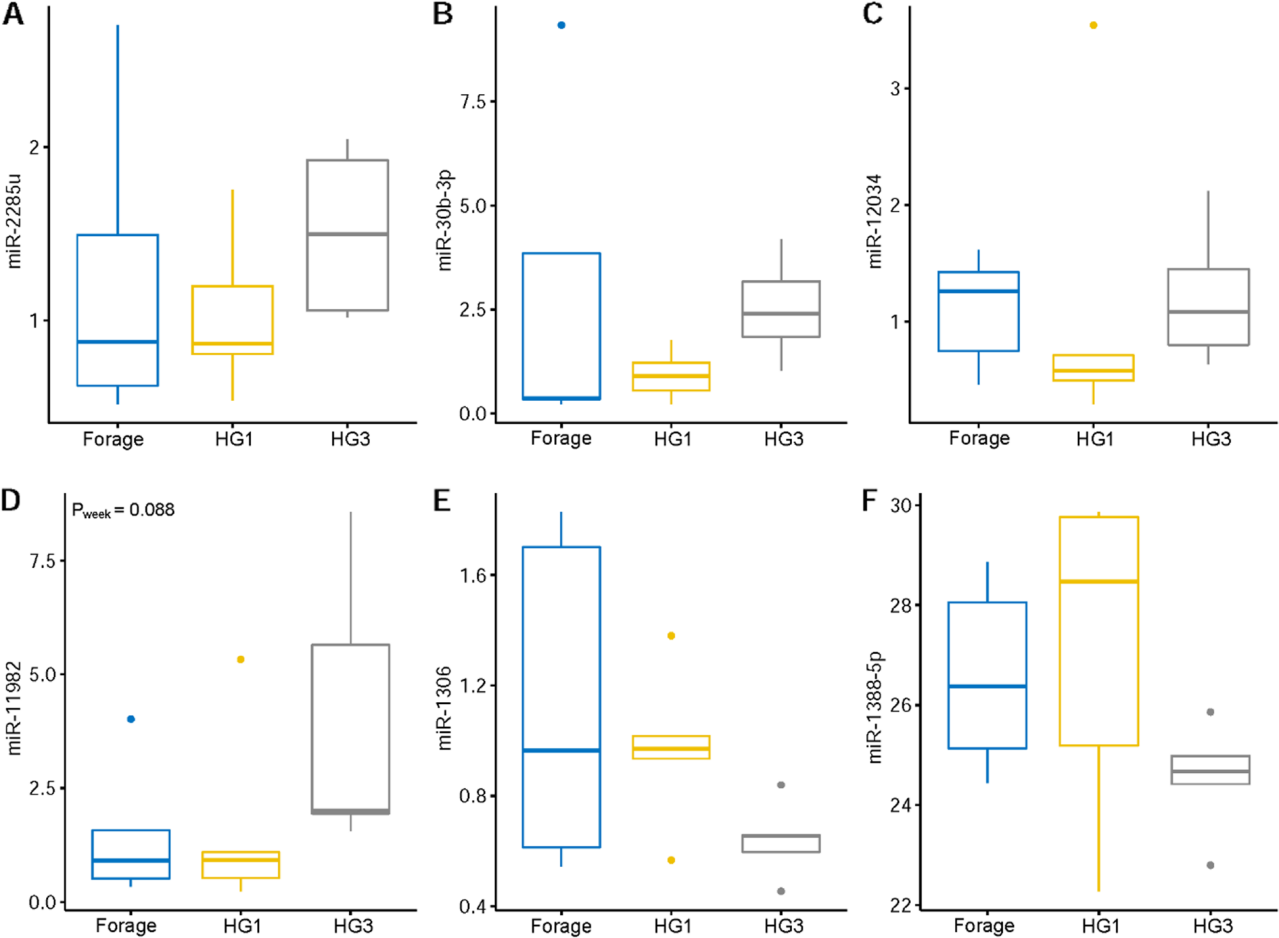
RT-qPCR validation of selected miRNAs as potential candidate biomarkers in leucocytes. Targets were selected based on their expression in plasma and are the same as in Fig. 6. MiR-103 and miR-107 were used as internal standard for normalization of all expression levels. HG1 = First week of high-grain feeding, HG3 = Third week of high-grain feeding

MicroRNA bta-miR-2285u had a higher relative expression when cows were fed FD (*P* = 0.04), while the relative expression was reduced as the cows transitioned to HG feeding throughout the weeks (Fig. 6a). On the contrary, in leucocytes the relative expression of bta-miR-2285u increased in HG3, and differential expression was visible (Fig. 7a). When compared to forage feeding, microRNA bta-miR-30b-3p had a higher expression in the first week of high-grain feeding (HG1) (*P* = 0.10) in plasma, which is in line with our NGS data results (Fig. 6b). In leucocytes, miR-30b-3p recorded a relatively higher expression in forage-based feeding in comparison to plasma (Fig. 7b). Bta-miR-12034 (Figs. 6c and 7c) only showed a tendency for differential expression between HG1 and HG3 (*P* = 0.10). For miR-11982 (Fig. 6d), we identified a tendency for higher expression in HG3 with (*P* = 0.09) (Fig. 7d). Bta-miR-1306 (Fig. 6e) had a relative high expression in forage-based feeding, with a greater expression in HG1 (*P* = 0.03). The expression of miR-1306 was higher in forage-based feeding than in HG feeding (Fig. 7e). Bta-miR-1388-5p (Fig. 6f) had no significant differential expression, but a tendency towards lower expression with HG feeding and in leucocytes (*P* = 0.10) (Fig. 7f). Bta-miR-1388-5p had a higher expression after HG3 (3 weeks of high grain feeding).

**Fig. 8:**
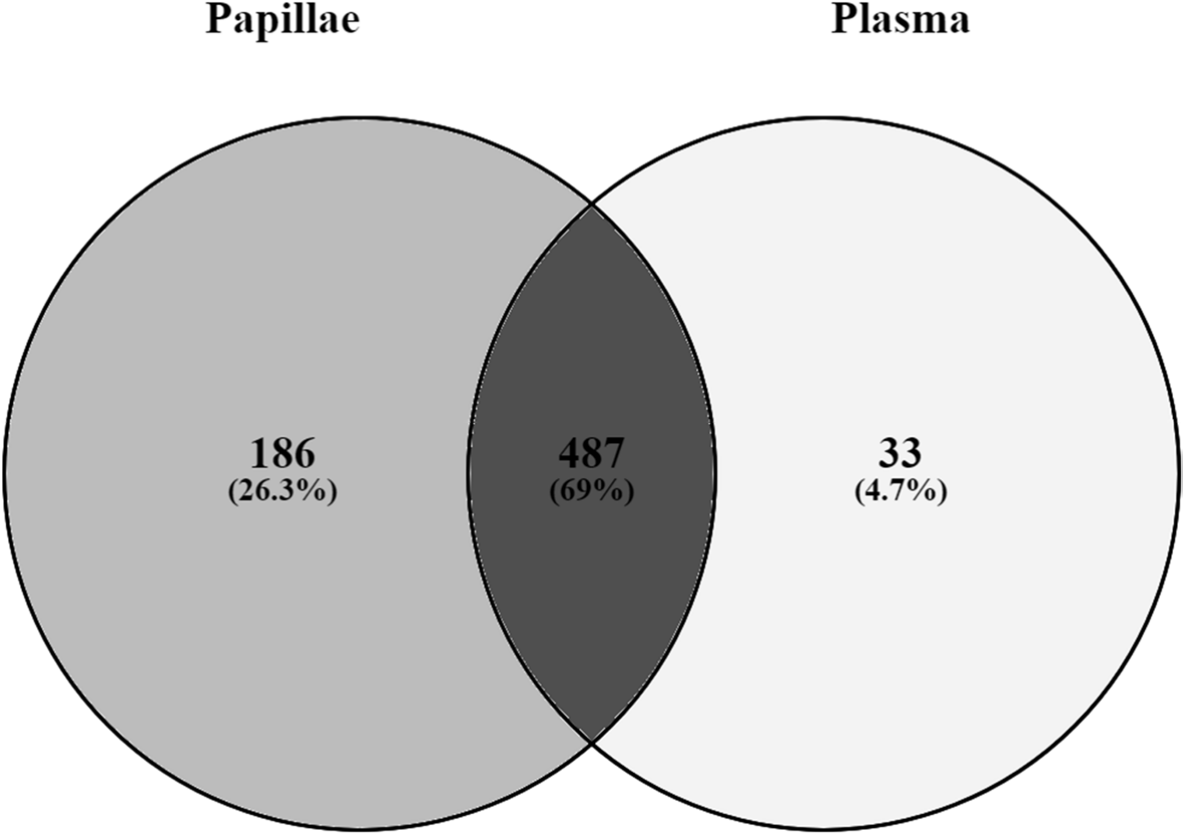
Venn diagram of miRNAs in papillae and plasma. 86 miRNAs were exclusively expressed in papillae while 33 miRNAs were exclusively expressed in plasma and a total of 487 miRNAs were shared between papillae and plasma

To further understand the significance of our results in plasma, we compared the expression profile of miRNAs in plasma to the expression profile of miRNAs in rumen papillae from the same cows, which were sampled for a companion paper [40]. In total, we identified 673 miRNAs in papillae and 520 miRNAs in plasma. There were 487 miRNAs shared between rumen papillae and blood plasma, accounting for 69% of all miRNAs. 186 miRNAs were found to be expressed only in papillae, while 33 miRNAs were found to be expressed exclusively in plasma (Fig. 8). The top 25 miRNAs were shared in blood plasma and rumen papillae. MiR-21-5p, miR-27b, let-7f, and let-7a-5p were among the top ten most expressed miRNAs in our list (Table1).

The miRNAs miR-2285u, miR-30b-3p, miR-12034, miR-11982, and miR-1306 that were chosen for in-depth analysis in plasma were also found to be expressed in papillae.

## Discussion

SARA has been investigated extensively in cattle, but viable systemic biomarkers have yet to be discovered. In our study, SARA was experimentally induced by feeding a HG diet. The aim of this study was to perform a systematic identification of miRNA profiles within blood (plasma and leucocytes), with the goal of evaluating them as possible candidate biomarkers in cows exposed to SARA.

The SARA is typically considered a metabolic disorder occurring within the rumen. This condition induces changes in the rumen microbiota and epithelium, increasing the leakage of microbial-derived toxins into the systemic circulation which in turn induce systemic inflammation, mainly via TLR-4/CD14-pathway [60]. The rumen epithelium is recognized to have a significant role in both metabolism and uptake of digested nutrients that are transported to peripheral tissues through the blood. Some evidence suggests that the process involves in impairment of the rumen epithelium's barrier function as a result of high luminal osmolality, which can result in swelling and rupture of ruminal papillae after feeding HG based diets, despite the lack of a complete understanding of the mechanisms behind endotoxin translocation through stratified squamous epithelium [25]. Pathogens and dietary constraints both have the ability to impact the amount of intestinal endotoxins, hence endotoxin translocation via the gut is likely present. These challenges could lead to a considerable rise in intestinal endotoxin burden, which is already known to occur in ruminants during sub-acute ruminal acidosis [41]. Subacute ruminal acidosis has been linked to local inflammation events, and it is possible that local inflammation might open the way for larger dietary or microbe-derived molecules to freely diffuse through disrupted epithelial junctions of the rumen epithelium [37]. As a result, it's possible that the activation of an inflammatory cascade in the rumen epithelium during SARA leads to functional and structural changes like endocytosis of cell junctional proteins, epithelial apoptosis, and activation of myosin light chain kinase phosphorylation, which can lead to cytoskeletal contractions and increased permeability, as seen in inflamed intestinal tissues [60].

In our pilot study, when cows were fed a HG diet, there were more circulating miRNAs, with 63 miRNAs exclusively expressed with the HG diet. The HG diet had a considerable influence (*P* = 0.04) on miR-2285u expression (Fig. 6a). MiRNA bta-miR-2285 variants could be employed as a candidate biomarker for rumen health in cattle. Sun and colleagues discovered six rumen-specific miRNAs in their research: bta-miR-2285 s, bta-miR-6527, bta-miR-1434-3p, bta-miR-2387, bta-miR-2344, and bta-miR-615 [51]. They also labelled the bta-miR-2285 mutation as rumen-related, suggesting that the miRNAs discovered in this study could be potentially used as biomarker. With 6100 targeted transcripts, bta-miR-2285u was also recognized as an excellent candidate to serve as biomarker for SARA from our list of prospective candidate biomarkers. In our cow model, we observed 23 mutations of bta-miR-2285 in blood plasma, 60 mutations of bta-miR-2285 in leucocytes and 47 mutations of bta-miR-2285 in rumen papillae samples. We discovered that the miRNAs selected for further investigation were also found in papillae, implying that they could be secreted into the bloodstream from the ruminal epithelium. Circulating miRNAs could be released from cells into the blood or any other body fluid in either an active (secretion) or passive (membrane leaking) way [9, 28, 30]. A great deal of interest generated by circulating miRNAs relates to their involvement in the regulation of molecular pathways of recipient cells and their exceptional potential as easily accessible biomarkers of diseases and disorders [48]. MiR-2285 was found to be highly expressed in HG3, suggesting an association with SARA and therefore with stressful conditions for the rumen. This result is consistent with our companion paper, which showed that our model was effective in inducing SARA where the cows experienced severe SARA, as indicated by increased in time spent with a pH below 5.8 [45].

Muroya and colleagues showed that grazing on pasture affected the plasma miRNAs compared to grain-feeding in Japanese black cattle. In particular, they found bta-miR-30b-5p to be highly expressed in plasma of cattle given a high-grain diet as compared to grazing cattle [39]. We identified bta-miR-30b-3p (Fig. 6b) with a higher relative expression in high grain diet than in the forage-based diet, both via NGS and small RNA RT-qPCR. Therefore, we believe that bta-miR-30b-3p could be linked to high grain feeding and subsequently to SARA. Hence, bta-miR-30b-3p is an incredibly viable candidate to serve as a candidate biomarker for rumen health in cows at risk of developing SARA. It targets 4313 transcripts which implies that miR-30b-3p plays a crucial role in post-transcriptional regulation. There are several immune related genes targeted, such as *CD28*, which has 4 different suggested seed regions. CD28 is a T-cell co-receptor which leads to IL-6 production. Another target of miR-30b-3p is *CD160*, which is targeted by 3 sites. CD160 is a glycoprotein which is expressed on peripheral blood NK cells and CD8 T -lymphocytes with cytolytic effector activity (Table 3) [54]. Immunoglobulin superfamily member 6 (*IGSF6*), molecule like family member G (*CD300LG*), and sialomucin core protein 24 (*CD164*) were all linked to genes involved in immune response and had seed regions of 1, 2, 2, respectively. *CD164* encodes transmembrane sialomucin and cell adhesion molecule that regulates the proliferation, adhesion, and migration of hematopoietic progenitor cells. The encoded protein affects muscle organ development by interacting with the C-X-C chemokine receptor type 4 [5]. Summarizing our findings, we believe that the expression of bta-miR-30b-3p in plasma could be linked to the metabolic state of the cows.

In our study, also miR-1388-5p was identified among the list of the 12 differentially expressed (DE) miRNAs in plasma for forage against HG diets with a read count of 595 and therefore was further analysed. In a previous study by Wang and colleagues, miR-1388-5p was identified as one of the 25 core differentially expressed miRNAs in relation to the formation of the mammary gland development and the differentiation of epithelial cell terminals during lactation compared to non-lactation [57]. With two separate seed areas, bta-miR-1388-5p targets 2715 transcript with diverse immune related genes targeted, such as *CD84*, which contributes to the adaptive immune response. With seed regions of 1 each, *CD86* and *CD300LB* are also involved in adaptive immune response (Table 3) [54]. Therefore, miR-1388-5p was also considered as a potential candidate biomarker of SARA but could not be confirmed by small RNA RT-qPCR. Bta-miR-11982 (Fig. 6d) shows a lower expression during the forage feeding with a lower expression after HG3. Bta-miR-11982 (Fig. 7d) indicates a higher expression in HG3 diets with a p-value of 0.088. We were unable to confirm a regulation of bta-miR-12034 using RT-qPCR after feeding high-grain diets (Figs. 6c and 7c).

In a previous study of our group, we examined the effects of a high-grain diet on miRNA and mRNA expression of ruminal epithelial tissue [40]. We discovered miR-21-5p, miR-27b, let-7a-5p, let-7f, and miR-205 in the rumen papillae of cows, which were also found in the blood of cows in a previous study [23]. This is in line with the findings of the current study, in which we found that bta-miR-21-5p, bta-let-7f, and bta-let-7a-5p, as well as bta-miR-143 and bta-miR-26a, were among the 25 most expressed miRNAs in both plasma and papillae. Although they showed high read counts in plasma, they were not DE between forage and high-grain, therefore we did not analyse them further to determine their suitability as candidate biomarkers. These highly abundant miRNAs found in rumen papillae, plasma, and leucocytes in both feeding regimes in our study have previously been linked to immunity and environmental stress sensing in cows, bovine skeletal muscle development, connective tissue cell differentiation, and glucose and lipid metabolism regulation in mice, demonstrating their biological relevance in tissue development and immunity [19, 32, 38, 62].

In cattle, Ioannidis and Donadeu looked for possible biomarkers of tissue-function in plasma and blood cells and discovered 5 miRNAs (miR-486, miR-142-5p, miR-191, miR-92a, and miR-30e-5p) that are shared between plasma and blood cells [23]. Bovine miR-486, miR-92a, and miR-143 were also identified in our study from the list of the most expressed and shared miRNAs in plasma and leucocytes. Although, we found in our study that the top three most represented miRNAs, miR-451, miR-21-5p, and hsa-miR-451a, were the same in both plasma and leucocytes. During forage-based feeding, read counts were often low, however, some cows in our study on the FD had a higher read count than the rest. This could be related to the cows' specific genetic variations. Limitations of our pilot study are the relatively small sample size of four animals per analysed group. Therefore, there is the need for more research in affected cow herds to demonstrate that the expression of these miRNAs is constantly associated to cows that are suffering from SARA.

## Conclusion

The results of our pilot study demonstrate that dietary modifications affect the release and expression of miRNAs in systemic circulation, potentially influencing post-transcriptional gene expression in SARA-affected cows. MicroRNA bta-miR-30-3p was identified and selected to be a top candidate to serve as a biomarker for SARA.. Additionally, the mutation of bta-miR-2285 were also differentially expressed between the forage based diet and the SARA inducing high-grain diet, suggesting that they could be candidate biomarker for rumen health. Therefore, microRNAs bta-miR-30b-3p and bta-miR-2285u appear to be promising candidates and should be investigated further.

## Methods

### Cows and diet-induced SARA model

The cows used in this research are a subset from a larger experiment conducted at the research and training farm of the University of Veterinary Medicine Vienna, Austria and published by Rivera-Chacon et al. [45]. The study was approved by the Institutional Ethics and Animal Welfare Committee of the University of Veterinary Medicine Vienna and the Austrian national authority according to the law for animal experiments (protocol number: BMNWF-68.205/0003-V/3b/2019) and conducted in compliance with ARRIVE guidelines. In brief, we used four ruminal-cannulated non-lactating Holstein cows (average age of 11.30 ± 2.29 years, body weight of 909.75 ± 98.17 kg). Cows were fed first a diet of 100% forage and were transitioned after one week of adaptation to a 65% high-grain (HG) diet to induce SARA. Details of the diet composition and feeding protocol are given in [45]. The presence of SARA was confirmed using a ruminal pH threshold of 5.8 for an average duration of 320 min/d [59], whereby the ruminal pH was monitored every 15 min via Lethbridge Research Centre Ruminal pH Measurement System (LRCpH,Dascor Inc., CA, USA) [45]. After the study, cows were released on pasture at the research and training farm of the University of Veterinary Medicine Vienna.

### Blood sampling

Blood samples were collected in EDTA-coated tubes (Becton Dickinson, USA) from the jugular vein before the morning meal. To examine miRNA expression profiles, blood samples were obtained and analysed during forage feeding and at week one of the HG feeding (HG1). For validation of NGS results and further analysis additional blood samples from the same cows were obtained at week three of HG feeding (HG3).

Blood samples were centrifuged at 2,000 × g for 15 min at 4 °C to obtain 3 layers: pellet, buffy coat, and plasma. The buffy coat was utilized to evaluate cellular miRNA expression in leucocytes. To obtain cell-free plasma, the supernatant (plasma) was centrifuged a second time at 800 × g for 15 min at 4 °C. The cell-free plasma was employed to analyse free circulating miRNAs. All samples were stored at -80 °C until further processing.

### Total RNA Isolation

Total RNA from 400 µl plasma sample was extracted using NucleoSpin Plasma miRNA Kit (Macherey–Nagel, Germany) following the manufacturer´s protocol for isolation of small and large RNAs. Total RNA from leucocyte pellets was conducted using NucleoSpin miRNA kit from Macherey–Nagel taking 200 µl of each pellet sample as starting material. The RNA was eluted using 50 µl of RNase-free water. All samples were frozen and stored at -80 °C. Quality control was performed using a chip for small RNAs (Agilent, California, USA) in a Bioanalyzer instrument (Agilent 2100 Bioanalyzer, California, USA). The RNA concentration and wavelength peak were determined using a DeNovix spectrometer.

### Small RNA sequencing

The NEXTflex Small RNA-Seq Kit (Bioo Scientific) was used to prepare small RNA libraries from each matrix, which were then sequenced on an Illumina NovaSeq 6000 utilizing a 50-base single-end sequencing method by CeGaT GmbH (Tübingen). Illumina bcl2fastq (2.20) was used to demultiplex the sequencing reads, and Skewer was used to trim the adapters (version 0.2.2). Demultiplexed reads were analysed using sRNAbench [1] and reads with an average PHRED score below 20 were discarded. To identify bovine (bta) miRNAs and human (hsa) miRNA homologues, the analysis was run in genome mode with the cow genome (UMD3 1 mp) and miRBase 22 as a reference. Reads below 15 nucleotides were discarded before mapping and were not included in further analyses. Bowtie was used to map sequence reads against the miRNA library allowing two nucleotide mismatches. Data on raw sequencing can be found in the GEO database (GSE198854).

### Differential expression analysis of miRNAs and predictions of DEmiRNAs Target

Differential expression of miRNAs were calculated with DESeq2 [36] (version 1.30.1), using the model: Y = cow + diet. The DE miRNAs and genes were identified using the Wald test and by computing the appropriate contrasts. Differences in expression were considered significant at a Benjamini and Hochberg [2] corrected *P* ˂ 0.05. Differentially expressed (DE) miRNAs were defined as microRNAs with an adjusted *p* ≤ 0.05 and a |log2 (fold change) |≥ 1. TargetScan [53] and miRBase [13] were used to predict DE miRNA target genes based on miRNA sequences.

The heatmaps with hierarchical clustering were created using ggplot [58] in R [43], based on normalized counts of miRNAs from DESeq2 [36] (version 1.30.1), and the principal component analysis (PCA) plot was likewise visualized and adjusted using DESEq2 [36] (version 1.30.1) and ggplot [58] packages.

### Quantification of selected miRNAs by RT-qPCR

Quantification of selected DE miRNA was done by a poly-A-technique according to the manufacturer’s protocol (miRNA 1st-Strand cDNA Synthesis Kit, Agilent Technologies). Therefore, 112 ng of total RNA was polyadenylated for 30 min at 37 °C using the 5 × Poly A Polymerase buffer, 10 mM of rATP and *E.coli* Polymerase A (2U/µL) and then terminated by 95 °C for 5 min in a total volume of 10 µL. In a second step, 10 µL of the elongated miRNA was reverse transcribed using the 10 × AffinityScript RT buffer, 10 µM of RT Adapter primer, 100 mM of dNTP mix and the AffinityScript RT/RNAse block enzyme mixture in a 20 µL volume. After incubation for 5 min at 55 °C and 15 min at 25 °C, reverse transcription (RT) was carried out at 42 °C for 30 min. Heating step at 95 °C for 5 min led to the inactivation of RT. Following reverse transcription, RT-qPCR was performed on a ViiA 7 Real-Time PCR System (Thermo Fisher Scientific, Waltham, USA) with three technical replicates per sample using 6.25 µL of 2 × miRNA QPCR Master Mix (Agilent Technologies), 0.5 µL of Universal Reverse Primer (3.125 µM, Agilent Technologies), 0.5 µL of specific small RNA primers (3.125 µM) and 3.5 ng of template in a total volume of 12.5 µL. Small RNA primers for the RT-qPCR were designed based on the sequence information from miRBase (Table 5). The amplification program consisted of the initial denaturation step at 95 °C for 10 min, followed by 45 cycles of 95 °C for 10 s, primer annealing at the optimal temperature for each specific primer (Table 5), and elongation at 72 °C for 30 s. Melting curve analysis was carried out for the specificity of the PCR amplification. Each qPCR assay was performed using negative template control and RT minus controls. MiR-103 (5’-AGCAGCATTGTACAGGGCTATGA-3’) and miR-107 (5’-CAAAGTGCTTACAGTGCAGGTAG-3’) served as reference miRNAs [55]. Their stable expression in bovine plasma and blood leukocytes were checked. Relative expression was calculated by the ΔΔCT-method [35] and has been described in detail, previously [27]. In short, different RNA content of the individual samples was corrected by the geometric mean of the CT values from the reference miRNAs (ΔCT). The miRNA expression was calculated relative to the individual values per each cow on the forage-based diet as baseline feeding (ΔΔCT). The results are shown as mean of the relative expression 2^−ddCT^. Overall effects were checked by ANOVA in R. Group differences were calculated by a Kruskal–Wallis test and were considered significant with *P* < 0.05.

**Table 5:** Selected miRNA primers for qPCR

**Targets**	**Annealing temperature**
F-mir-11982:TTCGGCGCCACCACCCTGCGGGT (23 bp)	60
F-mir-1388-5p: AGGACTGTCCAACCTGAGAAT (21 bp)	58
F-mir-12034: CCCCGGGGAGCCCGGCGGT (19 bp)	57.5
F-mir-2285u: GAAAAACCCGAACGAACTTT (20 bp)	57.5
F2-mir-30b-3p: TTCATTTGTAGGTGGAGGGTC (21 bp)	57.5
F-mir-1306: CCACCTCCCCTGCAAACGTCC (21 bp)	60

## Supplementary Information

**Additional file 1: Supplementary Table 1**. 25 Differentially expressed (DE) miRNAs in leucocytes (FDR < 0.05).

**Additional file 2: Supplementary Table 2**. 63 miRNAs expressed in every cow fed a high grain diet but not in each cow on a forage-based diet are listed below, along with their read counts (rpm) in plasma.

**Additional file 3: Supplementary Table 3**. 34 miRNAs expressed in every cow fed a high grain diet but not in each cow on a forage-based diet are listed below, along with their read counts (rpm) in leucocytes.

## Data Availability

This published manuscript, its additional information files, and publicly accessible repositories contain all data created or analysed during this investigation. The raw fastq files of the sequence data were submitted to the NCBI Gene Expression Omnibus (GEO) repository under the accession number GSE198854.

## Abbreviations

3’-UTR: 3’ Untranslated region
bp: Base pairs
Ca: Calcium
Cl: Chloride
Ct: Cycle threshold
DMI: Dry matter intake
FD: Forage based diet
HG: High grain diet
K: Potassium
LBP: Lipopolysaccharide-binding protein
LPS: Lipopolysaccharide
miRNAs: MicroRNAs
Na: Sodium
PCA: Principal component analysis
rpm: Reads per million
SARA: Subacute rumen acidosis
SCFA: Short chain fatty acids
