# Supplementary material for: Evaluation of circulating microRNA profiles in blood as potential candidate biomarkers in a subacute ruminal acidosis cow model - a pilot study
Source: BMC Genomics. 2023 Jun 16;24:333. doi: 10.1186/s12864-023-09433-y (PMC10273741; doi:10.1186/s12864-023-09433-y)
Supplement: Supplementary file 1 — Additional file 1: Supplementary Table 1. 25 Differentially expressed (DE) miRNAs in leucocytes (FDR < 0.05). [file 12864_2023_9433_MOESM1_ESM.docx]

**Supplement**

**Supplementary Table 1**: 25 Differentially expressed (DE) miRNAs in leucocytes (FDR < 0.05)

| **miRNA** | **baseMean** | **log_2_FoldChange** | **lfcSE** | **stat** | **pvalue** | **padj** |
| --- | --- | --- | --- | --- | --- | --- |
| hsa-miR-374a-5p | 19431 | -0.93936 | 0.150218 | -6.2533 | 4.02E-10 | 9.38E-08 |
| bta-miR-374a | 19431 | -0.93936 | 0.150218 | -6.2533 | 4.02E-10 | 9.38E-08 |
| bta-miR-3613a | 3045.902 | -1.01637 | 0.169161 | -6.0083 | 1.87E-09 | 2.92E-07 |
| bta-miR-146a | 50333.9 | -0.83635 | 0.155403 | -5.3818 | 7.37E-08 | 8.61E-06 |
| bta-miR-32 | 35648.89 | -0.78323 | 0.160148 | -4.89069 | 1.00E-06 | 9.38E-05 |
| hsa-miR-374b-3p | 536.3687 | -0.97482 | 0.225921 | -4.31486 | 1.60E-05 | 0.001243 |
| bta-miR-27a-3p | 78091.04 | -0.6445 | 0.151523 | -4.2535 | 2.10E-05 | 0.001404 |
| bta-miR-10225a | 95.27181 | 1.74033 | 0.414619 | 4.197419 | 2.70E-05 | 0.001576 |
| bta-miR-2285bf | 9382.906 | -0.65978 | 0.162768 | -4.0535 | 5.05E-05 | 0.002618 |
| bta-miR-29b | 5604.186 | -0.58221 | 0.15176 | -3.83635 | 0.000125 | 0.005302 |
| hsa-miR-29b-3p | 5604.186 | -0.58221 | 0.15176 | -3.83635 | 0.000125 | 0.005302 |
| bta-let-7b | 40551.81 | 0.545145 | 0.14826 | 3.67694 | 0.000236 | 0.007463 |
| has-let-7b-5p | 40551.81 | 0.545145 | 0.14826 | 3.67694 | 0.000236 | 0.007463 |
| bta-miR-135b | 335.01 | -0.9016 | 0.242803 | -3.7133 | 0.000205 | 0.007463 |
| bta-miR-142-3p | 36886.9 | -0.60871 | 0.165724 | -3.67302 | 0.00024 | 0.007463 |
| bta-miR-423-5p | 23861.88 | 0.505339 | 0.143734 | 3.515784 | 0.000438 | 0.012797 |
| bta-miR-484 | 31280.37 | 0.503897 | 0.147199 | 3.423237 | 0.000619 | 0.016999 |
| bta-miR-143 | 44604.85 | -0.56784 | 0.171302 | -3.31486 | 0.000917 | 0.023788 |
| bta-miR-10a | 8442.928 | -0.5195 | 0.159838 | -3.25016 | 0.001153 | 0.028349 |
| bta-miR-147 | 320.5609 | -0.93074 | 0.291552 | -3.19238 | 0.001411 | 0.032948 |
| bta-miR-22-3p | 95129.39 | -0.4513 | 0.142415 | -3.16887 | 0.00153 | 0.034032 |
| bta-miR-195 | 990.3367 | -0.56435 | 0.179876 | -3.13742 | 0.001704 | 0.03618 |
| bta-miR-29c | 9977.337 | -0.44058 | 0.146236 | -3.01281 | 0.002588 | 0.048352 |
| hsa-miR-29c-3p | 9977.337 | -0.44058 | 0.146236 | -3.01281 | 0.002588 | 0.048352 |
| bta-miR-24-3p | 146896.6 | -0.4251 | 0.14054 | -3.02476 | 0.002488 | 0.048352 |
