# Supplementary material for: Evaluation of circulating microRNA profiles in blood as potential candidate biomarkers in a subacute ruminal acidosis cow model - a pilot study
Source: BMC Genomics. 2023 Jun 16;24:333. doi: 10.1186/s12864-023-09433-y (PMC10273741; doi:10.1186/s12864-023-09433-y)
Supplement: Supplementary file 2 — Additional file 2: Supplementary Table 2. 63 miRNAs expressed in every cow fed a high grain diet but not in each cow on a forage-based diet are listed below, along with their read counts (rpm) in plasma. [file 12864_2023_9433_MOESM2_ESM.docx]

**Supplementary Table 2**: 63 miRNAs expressed in every cow fed a high grain diet but not in each cow on a forage-based diet are listed below, along with their read counts (rpm) in plasma.

| **Plasma** | | | | | | | | |
| --- | --- | --- | --- | --- | --- | --- | --- | --- |
| **microRNA** | **1** | **2** | **3** | **4** | **1** | **2** | **3** | **4** |
|  | **Forage** | | | | **High grain** | | | |
| bta-miR-10175-5p | 0 | 0 | 134 | 0 | 73 | 2 | 51 | 148 |
| bta-miR-11971 | 0 | 195 | 285 | 5 | 121 | 4 | 2 | 52 |
| bta-miR-11973 | 0 | 0 | 2 | 0 | 224 | 2 | 2 | 66 |
| bta-miR-11982 | 3 | 313 | 0 | 545 | 904 | 71 | 7 | 298 |
| bta-miR-1247-5p | 0 | 2 | 0 | 6 | 5 | 8 | 29 | 2 |
| bta-miR-138 | 0 | 370 | 0 | 336 | 212 | 6 | 2 | 363 |
| bta-miR-1388-5p | 0 | 2 | 0 | 0 | 265 | 47 | 9 | 272 |
| bta-miR-17-3p | 9 | 3 | 2 | 0 | 19 | 15 | 23 | 115 |
| bta-miR-196a | 0 | 2 | 2 | 3 | 9 | 59 | 8 | 2 |
| bta-miR-196b | 5 | 5 | 0 | 6 | 375 | 39 | 18 | 49 |
| bta-miR-206 | 4 | 2 | 0 | 735 | 49 | 29 | 47 | 198 |
| bta-miR-2284w | 3 | 0 | 8 | 10 | 13 | 13 | 20 | 251 |
| bta-miR-2284y | 0 | 8 | 2 | 3 | 95 | 10 | 9 | 14 |
| bta-miR-2285av | 0 | 8 | 2 | 3 | 95 | 10 | 9 | 14 |
| bta-miR-2285b | 3 | 6 | 6 | 0 | 9 | 18 | 82 | 41 |
| bta-miR-2285j | 0 | 3 | 6 | 6 | 57 | 10 | 60 | 112 |
| bta-miR-2285u | 0 | 0 | 0 | 165 | 2 | 4 | 2 | 432 |
| bta-miR-2411-3p | 0 | 0 | 2 | 0 | 55 | 2 | 4 | 103 |
| bta-miR-2448-3p | 3 | 0 | 2 | 0 | 3 | 4 | 5 | 118 |
| bta-miR-2454-3p | 0 | 584 | 0 | 0 | 228 | 78 | 89 | 31 |
| bta-miR-2457 | 0 | 4 | 0 | 3 | 42 | 3 | 8 | 8 |
| bta-miR-29d-5p | 0 | 377 | 8 | 327 | 262 | 93 | 19 | 32 |
| bta-miR-301b | 0 | 0 | 72 | 214 | 146 | 4 | 6 | 46 |
| bta-miR-30b-3p | 0 | 0 | 0 | 0 | 240 | 3 | 5 | 116 |
| bta-miR-324 | 0 | 0 | 0 | 0 | 134 | 4 | 2 | 5 |
| bta-miR-338 | 0 | 7 | 0 | 4 | 294 | 12 | 61 | 111 |
| bta-miR-33a | 0 | 3 | 0 | 4 | 4 | 73 | 9 | 45 |
| bta-miR-345-5p | 2 | 416 | 0 | 2 | 2 | 12 | 17 | 79 |
| bta-miR-369-5p | 0 | 0 | 5 | 0 | 4 | 3 | 11 | 2 |
| bta-miR-376a | 2 | 0 | 0 | 0 | 5 | 5 | 12 | 3 |
| bta-miR-378d | 0 | 4 | 2 | 3 | 6 | 4 | 8 | 11 |
| bta-miR-381 | 0 | 4 | 3 | 2 | 10 | 9 | 15 | 10 |
| bta-miR-410 | 3 | 3 | 0 | 4 | 7 | 8 | 16 | 12 |
| bta-miR-432 | 2 | 0 | 5 | 2 | 14 | 11 | 16 | 2 |
| bta-miR-433 | 3 | 3 | 0 | 0 | 9 | 9 | 15 | 7 |
| bta-miR-455-5p | 0 | 271 | 2 | 5 | 5 | 8 | 12 | 14 |
| bta-miR-483 | 2 | 6 | 2 | 0 | 12 | 4 | 16 | 14 |
| bta-miR-485 | 0 | 3 | 2 | 2 | 2 | 2 | 12 | 5 |
| bta-miR-493 | 0 | 3 | 2 | 2 | 5 | 8 | 11 | 10 |
| bta-miR-584 | 0 | 8 | 6 | 5 | 5 | 7 | 25 | 7 |
| bta-miR-628 | 7 | 9 | 0 | 5 | 399 | 16 | 21 | 136 |
| bta-miR-6517 | 2 | 289 | 4 | 0 | 6 | 9 | 3 | 126 |
| bta-miR-655 | 0 | 4 | 5 | 4 | 143 | 5 | 12 | 10 |
| bta-miR-656 | 3 | 4 | 0 | 3 | 4 | 6 | 13 | 5 |
| bta-miR-664b | 3 | 2 | 2 | 0 | 107 | 124 | 42 | 4 |
| bta-miR-7180 | 2 | 0 | 0 | 0 | 121 | 4 | 4 | 70 |
| bta-miR-7860 | 0 | 2 | 3 | 0 | 4 | 5 | 2 | 61 |
| bta-miR-877 | 0 | 0 | 288 | 336 | 440 | 79 | 3 | 232 |
| hsa-let-7f-1-3p | 0 | 2 | 0 | 3 | 10 | 5 | 7 | 6 |
| hsa-let-7f-2-3p | 2 | 4 | 2 | 0 | 100 | 5 | 10 | 7 |
| hsa-miR-138-5p | 0 | 370 | 0 | 336 | 212 | 6 | 2 | 363 |
| hsa-miR-196a-5p | 0 | 2 | 2 | 3 | 9 | 59 | 8 | 2 |
| hsa-miR-196b-5p | 5 | 5 | 0 | 6 | 375 | 39 | 18 | 49 |
| hsa-miR-23a-5p | 0 | 0 | 0 | 236 | 330 | 52 | 57 | 212 |
| hsa-miR-29c-5p | 0 | 377 | 8 | 327 | 262 | 93 | 19 | 32 |
| hsa-miR-30c-2-3p | 0 | 4 | 4 | 2 | 11 | 9 | 16 | 10 |
| hsa-miR-324-5p | 0 | 0 | 0 | 0 | 134 | 4 | 2 | 5 |
| hsa-miR-376a-3p | 2 | 0 | 0 | 0 | 5 | 5 | 12 | 3 |
| hsa-miR-433-3p | 3 | 3 | 0 | 0 | 9 | 9 | 15 | 7 |
| hsa-miR-483-3p | 2 | 6 | 2 | 0 | 12 | 4 | 16 | 14 |
| hsa-miR-485-5p | 0 | 3 | 2 | 2 | 2 | 2 | 12 | 5 |
| hsa-miR-628-5p | 7 | 9 | 0 | 5 | 399 | 16 | 21 | 136 |
| hsa-miR-99b-3p | 0 | 0 | 5 | 2 | 5 | 9 | 15 | 7 |
