# Supplementary material for: Evaluation of circulating microRNA profiles in blood as potential candidate biomarkers in a subacute ruminal acidosis cow model - a pilot study
Source: BMC Genomics. 2023 Jun 16;24:333. doi: 10.1186/s12864-023-09433-y (PMC10273741; doi:10.1186/s12864-023-09433-y)
Supplement: Supplementary file 3 — Additional file 3: Supplementary Table 3. 34 miRNAs expressed in every cow fed a high grain diet but not in each cow on a forage-based diet are listed below, along with their read counts (rpm) in leucocytes. [file 12864_2023_9433_MOESM3_ESM.docx]

**Supplementary Table 3**: 34 miRNAs expressed in every cow fed a high grain diet but not in each cow on a forage-based diet are listed below, along with their read counts (rpm) in leucocytes.

| **Leucocytes** | | | | | | |
| --- | --- | --- | --- | --- | --- | --- |
| **microRNA** | **1** | **3** | **4** | **1** | **3** | **4** |
|  | **Forage** | | | **High grain** | | |
| bta-miR-10179-5p | 0 | 18 | 3 | 3 | 16 | 12 |
| bta-miR-11994 | 74 | 79 | 0 | 70 | 54 | 27 |
| bta-miR-12064 | 15 | 8 | 0 | 9 | 15 | 3 |
| bta-miR-1434-5p | 4 | 23 | 0 | 2 | 11 | 5 |
| bta-miR-1814c | 13 | 10 | 0 | 17 | 24 | 6 |
| bta-miR-1949 | 12 | 14 | 0 | 16 | 22 | 13 |
| bta-miR-219 | 20 | 47 | 0 | 23 | 13 | 9 |
| bta-miR-219b-3p | 49 | 35 | 0 | 28 | 31 | 16 |
| bta-miR-2284e | 16 | 12 | 0 | 7 | 10 | 6 |
| bta-miR-2284p | 58 | 23 | 0 | 36 | 43 | 26 |
| bta-miR-2285ab | 30 | 28 | 0 | 17 | 5 | 4 |
| bta-miR-2285be | 9 | 16 | 0 | 4 | 4 | 2 |
| bta-miR-2285bl | 25 | 57 | 0 | 17 | 13 | 7 |
| bta-miR-2285bs | 25 | 28 | 0 | 6 | 8 | 8 |
| bta-miR-2285cj | 32 | 32 | 0 | 29 | 12 | 7 |
| bta-miR-2285x | 16 | 14 | 0 | 2 | 7 | 4 |
| bta-miR-2289 | 2 | 10 | 0 | 9 | 4 | 6 |
| bta-miR-2300a-5p | 13 | 6 | 0 | 9 | 16 | 5 |
| bta-miR-2310 | 13 | 10 | 0 | 17 | 24 | 6 |
| bta-miR-2311 | 16 | 12 | 0 | 16 | 5 | 5 |
| bta-miR-2370-3p | 6 | 17 | 0 | 13 | 11 | 19 |
| bta-miR-2415-5p | 21 | 13 | 0 | 8 | 11 | 2 |
| bta-miR-2425-3p | 17 | 0 | 14 | 19 | 4 | 50 |
| bta-miR-2463 | 24 | 5 | 0 | 30 | 12 | 12 |
| bta-miR-2469 | 2 | 13 | 0 | 4 | 2 | 2 |
| bta-miR-29e | 15 | 26 | 0 | 7 | 3 | 2 |
| bta-miR-383 | 22 | 29 | 0 | 12 | 5 | 11 |
| bta-miR-545-5p | 51 | 48 | 0 | 30 | 15 | 3 |
| bta-miR-6533 | 17 | 5 | 0 | 17 | 9 | 3 |
| bta-miR-7862 | 0 | 2 | 0 | 12 | 5 | 2 |
| bta-miR-940 | 7 | 0 | 0 | 10 | 3 | 6 |
| hsa-miR-193b-5p | 7 | 5 | 0 | 11 | 13 | 2 |
| hsa-miR-363-5p | 2 | 7 | 0 | 5 | 13 | 5 |
